# Rationale, design and organization of the delayed antibiotic prescription (DAP) trial: a randomized controlled trial of the efficacy and safety of delayed antibiotic prescribing strategies in the non-complicated acute respiratory tract infections in general practice

**DOI:** 10.1186/1471-2296-14-63

**Published:** 2013-05-19

**Authors:** Mariam de la Poza Abad, Gemma Mas Dalmau, Mikel Moreno Bakedano, Ana Isabel González González, Yolanda Canellas Criado, Silvia Hernández Anadón, Rafael Rotaeche del Campo, Pere Torán Monserrat, Antonio Negrete Palma, Guillem Pera, Eulàlia Borrell Thió, Carl LLor, Paul Little, Pablo Alonso Coello

**Affiliations:** 1 Centro de Atención Primaria Doctor Carles Ribas, Barcelona, Spain; 2 Iberoamerican Cochrane Centre, Biomedical Research Institute Sant Pau (IIB Sant Pau), Sant Antonio Ma Claret 167, Barcelona, 08025, Spain; 3 Centro de Salud Irutzun, Irurtzun, Spain; 4 Centro de Salud Vicente Muzas, Madrid, Spain; 5 Centro de Salud Monóvar, Madrid, Spain; 6 Centro de Atención Primaria Jaume I, Tarragona, Spain; 7 Centro de Salud Alza Osakidetza, Grupo Kronikgune sobre gestión del conocimiento, Donostia, Spain; 8 Grupo de Medicina Basada en la Evidencia de SemFYC, Madrid, Spain; 9 Unitat de Suport a la Recerca Metropolitana Nord, Institut Universitari d’Investigació en Atenció Primària Jordi Gol (IDIAP Jordi Gol), Santa Coloma de Gramenet, Spain; 10 Centro de Atención Primaria El Maresme, Mataró, Spain; 11 Centro de Atención Primaria Sant Roc, Badalona, Spain; 12 Aldermoor Health Centre, Aldermoor Close, Southhampton, UK

**Keywords:** Delayed antibiotic prescription, Respiratory infections, Family medicine, Pharyngitis, Acute tonsillitis, Rhinosinusitis, Acute bronchitis, Chronic obstructive pulmonary disease

## Abstract

**Background:**

Respiratory tract infections are an important burden in primary care and it’s known that they are usually self-limited and that antibiotics only alter its course slightly. This together with the alarming increase of bacterial resistance due to increased use of antimicrobials calls for a need to consider strategies to reduce their use. One of these strategies is the delayed prescription of antibiotics.

**Methods:**

Multicentric, parallel, randomised controlled trial comparing four antibiotic prescribing strategies in acute non-complicated respiratory tract infections. We will include acute pharyngitis, rhinosinusitis, acute bronchitis and acute exacerbation of chronic bronchitis or chronic obstructive pulmonary disease (mild to moderate). The therapeutic strategies compared are: immediate antibiotic treatment, no antibiotic treatment, and two delayed antibiotic prescribing (DAP) strategies with structured advice to use a course of antibiotics in case of worsening of symptoms or not improving (prescription given to patient or prescription left at the reception of the primary care centre 3 days after the first medical visit).

**Discussion:**

Delayed antibiotic prescription has been widely used in Anglo-Saxon countries, however, in Southern Europe there has been little research about this topic. The DAP trial wil evaluate two different delayed strategies in Spain for the main respiratory infections in primary care.

**Trial registration:**

This trial is registered with ClinicalTrials.gov, number http://NCT01363531.

## Background

Infectious diseases are among the most common complaints for family physicians. About 70% of these are respiratory tract infections, and the most common infections are acute bronchitis, pharyngitis and rhinitis [1]. Most of the respiratory tract infections are self-limiting, and according to recent systematic reviews, antibiotics have only a small effect on the course of most of these infections [2-10].

Besides, antibiotic treatment is no free of downsides including adverse events, increasing the costs for the same episode, and reinforcing beliefs about their usefulness. It has also been observed that antibiotic prescribing in respiratory tract infections correlates with a higher frequency of visits to the physician in the future, thus rising patients’ expectations about their use [11]. More importantly, though, the appearance and increase of bacterial antibiotic resistance is attributed mainly to the overuse of antibiotics [12]. This is a fundamental aspect since it may be increasingly more difficult to treat infections.

On the other hand, there is great variability in antibiotic prescribing among professionals, communities, and countries. Spain is one of the countries with the highest rate of antibiotic prescribing [13] and the rate of inappropriate antibiotic prescribing is also high, implying inappropriate use of resources [14,15].

Nevertheless, sometimes avoidance of antibiotic prescribing is still a challenge in patients with uncomplicated respiratory tract infections. Firstly, because it is not known which patients are at a higher risk of developing complications that, though uncommon, might be serious. Secondly, there are patients who wish and expect to be prescribed an antibiotic if symptoms do not subside [16], and physicians that overestimate the proportion of patients who expect be treated with antibiotics [17]. For these reasons, complete avoidance of antibiotic prescribing in patients with uncomplicated respiratory tract infections is not appropriate. 

Several studies have shown that the interventions aimed at reducing antibiotic prescribing may result in lower prevalence of resistant strains [12]. One of these interventions is the delayed prescription of antibiotics, in which the patient is prescribed an antibiotic that should be taken only in case of worsening of the symptoms or no improvement a few days after the visit. This strategy has been used in conditions where it may be difficult to differentiate between a viral and a bacterial etiology in primary care settings, such as respiratory tract infections and other infections such as conjunctivitis [18] or urinary tract infections [19].

A systematic review [20], concludes that delayed antibiotic prescription reduces antibiotic use without an increase in complications of these conditions, with little advantage compared with the non-prescription. This review shows how immediate compared to delayed antibiotic prescribing is associated with a consistently higher patient satisfaction. Additionally, other studies suggest that this strategy might reduce the number of visits [11,16] since patients can get rapid access to antibiotics in case of no improvement. Besides, as indicated by another systematic review [21] family doctors see this strategy as an intervention that may help them resolve uncertainties. Similarly, family doctors believe that reassures patients, providing them with control over their conditions and allowing them to reconcile with their expectations.

Despite the advantages seen in other parts of the world, no studies have evaluated the effectiveness and the acceptability of delayed prescription in Southern Europe. Only a single observational study [22] showed that this strategy might reduce antibiotic prescribing by 33%. For these reasons, we designed a multicenter, randomized clinical trial to assess delayed prescription in Spain.

The aim of this study is to establish the effectiveness of delayed antibiotic prescribing strategies versus antibiotic therapy and no therapy in terms of duration and severity of symptoms. Therefore, the study will establish the effectiveness of delayed antibiotic prescribing strategies versus antibiotic therapy and no therapy in terms of: antibiotic use at 30 days, satisfaction and belief in antibiotic effectiveness.

## Methods

Multicenter, randomized controlled clinical trial (Phase IV) comparing four therapy strategies for uncomplicated acute respiratory tract infections (clinicaltrials.gov, identifier NCT01363531).

### Randomization

Stratified by condition of interest (pharyngitis, rhinosinusitis, acute bronchitis, and exacerbations of chronic obstructive pulmonary disease). Centralized using a Web-based platform.

### Masking

Open-label study given the type of strategies used and given that an effect on their beliefs and the antibiotic use is expected.

### Experimental and control groups

Four treatment arms:

a) Group 1: immediate antibiotic therapy.

b) Group 2: no antibiotic therapy.

c) Group 3: delayed prescription by handing the prescription to the patient.

d) Group 4: delayed prescribing by collecting the prescription at the health care center from the third day after the initial visit.

The inclusion of no antibiotic and immediate antibiotic strategies is due to the need to evaluate their relative effectiveness compared with the delayed strategies. Potential differences among the delayed strategies are assessed by comparing the two delayed prescription strategies.

The choice of antibiotic therapy will depend on the specific respiratory tract condition and will be up to the physicians’ judgement, according to their usual clinical practice. Physicians will also handle symptomatic treatment and complications as needed.

### Inclusion and exclusion criteria

Patients over 18 years old with uncomplicated acute respiratory tract infections will be enrolled: acute pharyngitis, rhinosinusitis, acute bronchitis, and exacerbations of chronic bronchitis/mild to moderate chronic obstructive pulmonary disease. Patients with these infections will be included by the physicians as long as they are unsure of whether to use antibiotics or not. See details of the general and specific study criteria for each condition under [see Additional file 1].

### Study population and total number of patients

The sample size calculated is 600 patients. This is an estimate based on an average duration of 12 days (s.d. 6) for the main symptoms [16]. Considering a reduction of two days in the duration of the symptoms as a clinically relevant outcome, with a bilateral approximation, a sample of 600 patients will be able to detect this difference with an alpha error of 5% and a power of 80% (beta = 0.2). This estimate was calculated considering lower respiratory tract infections only. The project aims to be much broader (and therefore pragmatic, with an increase in the external validity at the conceptual phase), for this reason an exhaustive search will be carried out for all the conditions initially expected to be included (pharyngitis, rhinosinusitis, acute bronchitis, acute exacerbation of chronic bronchitis). The aim of this search is to obtain average values of the main endpoints defined in this project and to allow for standardization, and accordingly, a comparison (the variability that can be derived from the data obtained for the calculation has reached 50%, which indicates a high dispersion). It seems unlikely that the remaining conditions exceed that value, and generally, they will even tend to be lower. This allows avoiding the estimation of losses in the required total number of patients.

The patients will be equally distributed among the four evaluation groups to maximize power in the comparison. In addition, the four groups will be stratified in terms of the included conditions. Besides, focusing on the three groups except for the immediate antibiotic therapy, the 450 patients distributed in the three arms would allow to detect a difference of at least 13% in antibiotic use with the same indicated power (assuming that the figures for antibiotic use in the two delayed strategies are very similar: delayed strategy group 27% versus 14% in the group with no antibiotic therapy). Finally, in the comparison of four strategies for the variable *antibiotic use*, the study will have a 100% for antibiotic use as an outcome, since the percentage of antibiotic use in the antibiotic therapy arm is likely to exceed 90%. For the calculations we used Sample Power Release 2.0 software.

Criteria for withdrawals and expected analysis of withdrawals and dropouts:

•Serious adverse event

•Unsatisfactory therapeutic effect

•Patient clinical conditions prevent compliance with the protocol

•Protocol violation

•Informed consent withdrawal

Patients can choose to interrupt the medication any time during the course of the study. However, they will be followed up in the same way as the other patients. Analyses will follow the intention-to-treat principle, with patients being analysed in the groups to which they were randomized.

### Outcome variables

A) Main effectiveness endpoint

Duration and severity of symptoms. Patients will complete a validated symptom questionnaire daily [see Additional file 2] [16].

B) Secondary effectiveness endpoints

The following variables will be assessed:

Use of antibiotics: patients will be asked about antibiotic use in their visit at 30 days and this will be crosschecked with the corresponding pharmacy units in the sanitary areas.

Satisfaction with the strategy (questionnaire with a Likert scale to patients).

Belief in the effectiveness of antibiotics (questionnaire with a Likert scale to patients and health professionals).

Perception about safety and effectiveness (questionnaire with a Likert scale to health professionals).

C) Safety endpoints

Complications related to the infections will be registered during the first 30 days after randomization. These complications will be prospectively recorded by the physicians by means of a standardized questionnaire and will be reported in a maximum of 48 hours to the study clinical coordinator. This researcher will report to the Safety and Data Monitoring Committee, whose aim is to assess the safety of the evaluated strategies. The Clinical Event Allocation Committee - blinded to treatment allocation - will assess the primary outcomes. The decisions of the Assessment Committee will be used in all the statistical analyses of the primary outcome. This Committee will meet quarterly.

### Enrollment

Enrollment will be carried out competitively at the participating physicians’ clinics and is expected to take one year. The study will be carried out in five Spanish Autonomous Communities (Catalonia, Galicia, Basque Country, Navarre and Madrid).

### Data collection

#### Initial visit

Baseline data will be collected in the clinic by the physician or with the help of the nursing staff. To standardize data collection, participants will be trained by the coordinating center. Patients will receive information about the study and, if they are interested in participating, an informed consent to read and sign will be handed to them. A maximum length of 10–15 minutes is expected for the interview and the introduction of the data.

After the randomization, information on the strategy to which they have been allocated will be given to the participants, and they will be informed on the appropriate measures to take in case of worsening or no improvement. Besides, they will be given a diary with a validated questionnaire of symptoms for each condition [16] which they should complete while symptoms related to the respiratory condition are present [see Additional file 2]. The degree of satisfaction or concern with different aspects of the therapy will also be recorded in this diary.

#### Follow-up

Patients will be interviewed by telephone after 48 hours of their inclusion in the study. Monitoring calls are also planned at day 7, 15 and 22 if symptoms are still reported by the patient on the calls. After 30 days, patients will visit their physician and then will submit the completed diary. We will also include a one year follow-up.

All the study data will be entered in an electronic platform (http://www.grupopreada.com).

### Data analysis

In order to ensure their similarities, a baseline comparison of the treatment groups will be performed. If any clinically relevant differences are found, a multivariate analysis will also be performed to ensure the absence of biases resulting from them. Differences will be deemed significant if p < 0.05. The SPSS software version 21.0 will be used for the calculations.

The comparisons among the treatment groups will be as follows: The differences for categorical variables will be described using contingency tables (and the corresponding percentages); inferencing will be made by means of an exact Fisher test or Chi-square test. The Kruskall-Wallis test will be used for ordinal or quantitative variables with heteroscedasticity or abnormality issues. In case of significance, the decision on the groups will be based on the 2 × 2 Mann–Whitney test; no corrections will be applied for multiple comparisons, calculated the means and the higher and lower values for each group. For quantitative variables, the analysis of variance (ANOVA) test will be used, resorting to post-hoc in case of significance, thus showing the means and the standard deviations for each group.

#### Ethical issues

The protocol was approved by the Ethics Committee of Clinical Investigation *IDIAP Jordi Gol i Gurina* and by the Ethical Committees of Clinical Research in the corresponding health care areas and Spanish Agency of Medicines and Health Products. Each patient should give their written consent to participate in the study after being informed in intelligible language for him on the nature, scope and possible consequences of the trial. After consent is submitted, the patients will be randomized.

Data confidentiality will be ensured at all times, as it is stated in the researcher’s commitment sheet, as well as the compliance with current legislation regarding the protection of personal data. The clinical trial will be insured by the health provider to which the different participating health centers belong.

#### Other considerations

This is a clinical trial based on the outpatient setting, and no monetary compensation is given to either patients or researchers. Sponsorship is only reserved for the purposes of organization and implementation of the trial.

## Discussion

The main issue addressed in our study is the effectiveness of the delayed antibiotic prescribing strategy in uncomplicated respiratory infections. To achieve this we will compare two delayed prescription strategies with immediate antibiotic prescribing and no prescription. Specifically, symptom duration and severity, patient satisfaction, belief in the effectiveness of antibiotics and antibiotic use in the different strategies will be assessed.

There are two main types delayed strategies. One where patients are asked to return to the reception of the primary health center in order to collect the prescription, and another one in which the prescription is handed in the initial visit. The rationale for the former being that asking the patient to return could further reduce subsequent use of antibiotics [13,23].

Delayed antibiotic prescribing has been used not only for respiratory tract infections but also for other types of diseases [18,19]. So far, these studies have shown [20], that these strategies reduce antibiotic use. As to patients’ satisfaction, a qualitative study in patients with conjunctivitis showed that this strategy is usually well received [18] and another in women with urinary tract infections [19] showed positive perceptions too. In one of them there is the paradox that those patients who know that their condition is mild but still feel that they need them, given their strong belief in the need of a therapy [18]. As to the physicians [21], they highlight that these strategies can be of help in cases of uncertainty, since patients can gain fast access to antibiotics and in addition, the strategy reassures them too.

In our study the two delayed strategies will be compared with other alternatives and with each other. We also wish to find out whether they are effective reducing the number of new visits. Antibiotic prescribing in the clinic leads to a vicious circle in which the patient perceives that an antibiotic treatment is necessary, it increases the number of new visits for the same reason and increases the chance of requesting a new antibiotic in similar episodes in the future [11]. Consequently, we will evaluate differences among the delayed prescribing strategies in terms of the number of new visits and the immediate antibiotic prescribing strategy.

Delayed strategies are also criticized for an increased possibility of complications. In order to avoid them, it is crucial to bear in mind that these are not used in the case of patients presenting with supposedly bacterial infections, and reserved in the event of reasonable uncertainty. They should not be used in critically ill patients or those meeting criteria for admission [24].

Two systematic reviews have studied the use of delayed strategies and both have showed significant advantages. One of them [23] showed that for most of the symptoms no significant differences in duration have been observed compared with immediate antibiotic prescribing. In a more recent systematic review Spurling et al showed that delayed prescription significantly reduces antibiotic consumption [20].

Further studies [25] conclude that delayed prescribing is an acceptable option, reflected in small differences in terms of the resolution of the symptoms, and a considerable reduction in antibiotic use and in the confidence in its effectiveness. The number of randomized patients is still scarce and the results for some of the outcome are non conclusive. Nevertheless, some institutions such as NICE start to recommend it as an initial strategy for self-limiting respiratory tract infections [26].

Our study has several strengths but also some limitations. For example, the open-label design of the trial could imply a placebo effect favoring antibiotics. This effect is minimized given the structured approach that physicians will apply to support and increase the confidence in each strategy. In previous studies this placebo effect has been minimal in favor of antibiotics or has not been recorded [11,25]. Another limitation in our study is the absence of a unique consensus definition for respiratory tract infections. Due to this, we will use the definitions used in the main published cohort studies [27].

One of the important strengths of our study is the inclusion of all the main respiratory tract infections, considering for the first time the exacerbations of mild to moderate COPD. Besides, it will be the first study to compare two delayed strategies directly. The pragmatic character of the study increases the external validity of the results and this is of special interest to our health system priorities.

The confirmation of our hypothesis will likely reassure physicians about the effectiveness and safety of these delayed strategies. This fact could facilitate their implementation in our country and, thus, help optimizing antibiotic prescribing. We believe that our results will likely help to change patients’ perception about the role and need of antibiotics.

The impact of our trial is likely to be important since the available evidence in this field is still limited, and new larger studies, adequately designed and conducted, are warranted to clarify the effectiveness of these strategies. Similarly, including most of the uncomplicated respiratory infections will increase the external validity of the results. Comparing, for the first time, two delayed strategies will provide information on the possible differences within this modality. These characteristics make this study innovative and relevant to this field.

## Competing interests

The authors declare that they have no competing interests.

## Authors’ contributions

PAC conceived the study. PAC, MPA, RRC, CL, SHC, MMB, AIG, PTM, ANP, GP, EBT, IG, PL and GMD designed the study. PAC, MPA, GMD are responsible for conducting the trial. All other authors critically commented on the study design. MPA, CL, SHC, MMB, AIG, PTM, ANP, EBT, IG and all other authors who are family practitioners or nurses, belonging to the participant health centers, will recruit patients. All authors read critically and approved the final manuscript.

## Pre-publication history

The pre-publication history for this paper can be accessed here:

http://www.biomedcentral.com/1471-2296/14/63/prepub

## Supplementary Material

Additional file 1 Inclusion and exclusion criteria. Click here for file

Additional file 2 Diary (Symptom Questionnaire). Click here for file
