# Supplementary material for: Rationale, design and organization of the delayed antibiotic prescription (DAP) trial: a randomized controlled trial of the efficacy and safety of delayed antibiotic prescribing strategies in the non-complicated acute respiratory tract infections in general practice
Source: BMC Fam Pract. 2013 May 19;14:63. doi: 10.1186/1471-2296-14-63 (PMC3682866; doi:10.1186/1471-2296-14-63)
Supplement: Additional file 1 — Inclusion and exclusion criteria. [file 1471-2296-14-63-S1.pdf]

Additional file 1: Inclusion and exclusion criteria

The **general exclusion criteria** for the patients are as follows:

- Previous participation in the DAP trial.
- Patient is very affected or they have been very affected for a week (constantly)

- Patient with signs and symptoms suggestive of disease o serious affectation and/or of complications (particularly pneumonia, mastoiditis, peritonsillar abscess, peritonsillar cellulitis, intraorbital or intracranial complications).

- Patient at a risk high of serious complications due to a previous comorbidity. This includes serious conditions involving the heart, lungs, kidney, liver, or neuromuscular disorders, immunosuppression, cystic fibrosis.

- If the patient is older than 65 with acute cough and two or more of the following criteria or older than 80 with acute cough and one or more of the following criteria:
  - Admission in the previous year
  - Type I or II Diabetes
  - History of cardiac failure
  - Current use of oral corticosteroids.

Acute pharyngitis:

Same criteria as in Little PS, et al. are used BMJ 1997;314:722-7:

- Inclusion criteria: adults presenting to the family doctor with sore throat as the main symptom and 2 or 3 Centor criteria: (1) pharyngo-tonsillar exudate, (2) history of fever or feeling of dysthermia, (3) tender anterior cervical adenopathy and/or (4) absence of cough.

- Exclusion criteria: Other causes of sore throat (ulcers, aphthas or thrush), presence of the four Centor criteria, presence of only one or none of the Centor criteria, poor general condition, immunosuppression, antibiotic use in the previous two weeks, history of rheumatic fever, repeated pharyngotonsillitis (more than five episodes in the previous year), pregnant women and/or use of rapid antigen techniques in the visit.

Rhinosinusitis:

Same criteria as in other studies (Arroll B, et al. are used J Fam Pract 2002;51:324-8):

- Inclusion criteria: Acute inflammation of the mucose membrane in the nose and pharynx, for more than one week, with rhinitis as the main sign and at least one symptom or sign of the sinuses: purulent rhinorrhea or sinus pain.
- Exclusion criteria: Symptoms for less than a week, poor general condition, suspected pneumonia, antibiotic use in the previous two weeks and/or use of rapid C-reactive protein test in the visit. .

(*) Poor general condition: Patient is very affected or they have been very affected for a week.

Acute bronchitis:

Same criteria as in other studies (Little P, et al. are used JAMA 2005;293:3029-35 y Macfarlane J, et al. Thorax 2001;56:109-14):

- Inclusion criteria: Adults with uncomplicated acute disease presenting with cough as the main symptom and at least one symptom or sign of the lower respiratory tract: expectoration, chest pain, shortness of breath or wheezing.
- Exclusion criteria: Suspected pneumonia (crepitant rales, bronchial murmur, asymmetrical auscultation, tachypnea, vomiting and/or severe diarrhea), bronchial asthma, other acute respiratory tract or chronic diseases except for mild to moderate COPD (cystic fibrosis, tuberculosis), active cardiovascular disease, psychiatric diagnoses, dementia, poor general condition, institutionalized in care centers, prior antibiotic use in the previous two weeks, history of admission in the previous year due to respiratory tract infections and/or use of rapid C-reactive protein test in the visit.

Exacerbations of chronic bronchitis/mild to moderate chronic obstructive pulmonary disease:

- Inclusion criteria: > 40 years old, smokers or ex-smokers of over 10 packages-year, with one of the two following diagnoses: chronic bronchitis (cough for more than 3 months for more than 2 consecutive years), or chronic obstructive pulmonary disease diagnosed with spirometry less than 2 years before its last test (FEV_1/FVC <0.7% and FEV_1 50%), with an infectious exacerbation and one or two of the following Anthonisen criteria: increased expectoration volume and/or increased shortness of breath (Anthonisen NR, et al. Ann Intern Med 1987;106:196-204).
- Exclusion criteria: Purulent expectoration, no spirometry in the previous 2 years, severe COPD (FEV1 <50%), neoplasm, pregnancy, tracheotomy, poor general condition, antibiotic use in the previous two weeks, suspected pneumonia (crepitant rales, bronchial murmur, asymmetrical

auscultation, tachypnea, vomiting and/or severe diarrhea) and/or use of rapid C-reactive protein test in the visit.
