# Supplementary material for: Rationale, design and organization of the delayed antibiotic prescription (DAP) trial: a randomized controlled trial of the efficacy and safety of delayed antibiotic prescribing strategies in the non-complicated acute respiratory tract infections in general practice
Source: BMC Fam Pract. 2013 May 19;14:63. doi: 10.1186/1471-2296-14-63 (PMC3682866; doi:10.1186/1471-2296-14-63)
Supplement: Additional file 2 — Diary (Symptom Questionnaire). [file 1471-2296-14-63-S2.pdf]

Additional file 2: Diary (Symptom Questionnaire)

In the symptom diary the following aspects related to each condition will be collected:

FOR EACH SYMPTOM choose the degree of affectation in the last 24 hours, according to the following Table of codes:

0	Normal or does not upset me / does not hurt
1	Affectation is insignificant or affects me slightly / light pain
2	Mild or slightly significant affectation or affects me slightly or very little / moderate pain
3	Moderately significant affectation or affects me moderately / considerable pain
4	Significant affectation or affects me considerably / intense pain
5	Highly significant affectation or affects me highly significantly / very intense pain
6	Maximum affectation or affects me a great deal / unbearable pain

Color of secretions can also be specified in case of nasal mucus for rhinosinusitis, or of expectoration or phlegm for acute bronchitis and in the exacerbation of the COPD.

T	If transparent
Y	If light yellow
G	If green
O	If other
	Leave the box blank if no phlegm/mucus has been detected on that day.

Pharyngitis: 9 symptoms were recorded daily: feeling of fever, headache, general discomfort or pain, cough, sore throat, difficulty swallowing (solids or liquids), runny nose, difficulty sleeping and difficulty with activities of daily living. Each symptom will be scored following a 0-6 scale.

Diary of symptoms (Pharyngitis)

Please fill in the diary writing the numbers (according to Code Table) and indicating how you have felt in the last 24 hours.

Do this EVERY NIGHT (BEFORE YOU GO TO BED AND YOU HAVE TO START TODAY) until the day you feel fully recovered.

Example to help:
The person of the example below feels: very intense general discomfort or pain (5) and intense pain in sore throat (4).

Day	Ex	0	1	2	3	4	5	6	7	8	9	10	11	12	13	14	15	16	17	18	19	20	21	22	23	24	25	26	27	28	29	30
Feeling feverish	0																															
Headache	0																															
General discomfort or pain	5																															
Cough	0																															
Sore throat	4																															
Difficulty swallowing (solids or liquids)	0																															
Runny nose	0																															
Difficulty sleeping	0																															
Difficulty with activities of daily living	0																															

Figure 1

Bronchitis: 9 symptoms were recorded daily: feeling of fever, general discomfort or pain, cough, expectoration or phlegm (mucus when coughing), shortness of breath (suffocation, fatigue), pain when breathing (chest pain), chest breath sounds, difficulty sleeping and difficulty with activities of daily living. Each symptom (save for expectoration or phlegm) will be scored from 0 to 6. In case of expectoration or phlegm, their color will be assessed: transparent (T), light yellow clear (Y), green (V) or other (O).

Diary of symptoms (Bronchitis)

Please fill in the diary writing the numbers (according to Code Table) and indicating as you have felt in the last 24 hours.

Do this EVERY NIGHT (BEFORE YOU GO TO BED AND YOU HAVE TO START TODAY) until the day you feel fully recovered.

Example to help:
The person of the example below feels: very intense general discomfort or pain (5), significant affectation of shortness of breath (4) and yellow expectorations or phlegm (Y).

Day	**Ex**	0	1	2	3	4	5	6	7	8	9	10	11	12	13	14	15	16	17	18	19	20	21	22	23	24	25	26	27	28	29	30
Feeling feverish	0																															
General discomfort or pain	5																															
Cough	0																															
Expectoration or phlegm (mucus when coughing)	Y																															
Shortness of breath (suffocation, fatigue)	4																															
Pain when breathing (chest pain)	0																															
Chest breath sounds	0																															
Difficulty sleeping	0																															
Difficulty with activities of daily living	0																															

Figure 2

Exacerbation of chronic bronchitis or mild to moderate chronic obstructive pulmonary disease: 9 symptoms were recorded daily: feeling of fever, general discomfort or pain, cough, expectoration or phlegm (mucus when coughing), shortness of breath (suffocation, fatigue), pain when breathing (chest pain), chest breath sounds, difficulty sleeping and difficulty with daily life activities. Each symptom (except for expectoration or phlegm) will be scored from 0 to 6. In case of expectoration or phlegm, their color will be assessed.

Diary of symptoms (COPD)

Please fill in the diary writing the numbers (according to Code Table) and indicating how you have felt in the last 24 hours.

Do this EVERY NIGHT (BEFORE YOU GO TO BED AND YOU HAVE TO START TODAY) until the day you feel fully recovered.

Example to help:
The person of the example below feels: very intense general discomfort or pain (5), significant affectation of shortness of breath (4) and yellow expectorations or phlegm (Y).

Day	Ex	0	1	2	3	4	5	6	7	8	9	10	11	12	13	14	15	16	17	18	19	20	21	22	23	24	25	26	27	28	29	30
Feeling feverish	0																															
General discomfort or pain	5																															
Cough	0																															
Expectoration or phlegm (mucus when coughing)	Y																															
Shortness of breath (suffocation, fatigue)	4																															
Pain when breathing (chest pain)	0																															
Chest breath sounds	0																															
Difficulty sleeping	0																															
Difficulty with activities of daily living	0																															

Figure 3

Rhinosinusitis: 11 symptoms were recorded daily: feeling of fever, general discomfort or pain, headache, sudden pain in the face, pain in the face when touching, runny nose, nasal mucus color, cough, sore throat, difficulty sleeping, and difficulty with activities of daily living. Each symptom (save for nasal mucus color) will be scored from 0 to 6. In case of presence of nasal mucus color, the color will be assessed.

Diary of symptoms (Rhinosinusitis)

Please fill in the diary writing the numbers (according to Code Table), indicating how you have felt in the last 24 hours.

Do this EVERY NIGHT (BEFORE YOU GO TO BED AND YOU HAVE TO START TODAY) until the day you feel fully recovered.

Example to help:
The person of the example below feels: very intense general discomfort or pain (5), intense headache (4) and green nasal mucus (G).

Day	Ex	0	1	2	3	4	5	6	7	8	9	10	11	12	13	14	15	16	17	18	19	20	21	22	23	24	25	26	27	28	29	30
Feeling feverish	0																															
General discomfort or pain	5																															
Headache	4																															
Sudden pain in the face	0																															
Pain in the face when touching	0																															
Runny nose	0																															
Nasal mucus color	G																															
Cough	0																															
Sore throat	0																															
Difficulty sleeping	0																															
Difficulty with activities of daily living	0																															

Figure 4
